# ICEAGE (Incidence of Complications following Emergency Abdominal surgery: Get Exercising): study protocol of a pragmatic, multicentre, randomised controlled trial testing physiotherapy for the prevention of complications and improved physical recovery after emergency abdominal surgery

**DOI:** 10.1186/s13017-018-0189-y

**Published:** 2018-07-03

**Authors:** Ianthe Boden, Kate Sullivan, Claire Hackett, Brooke Winzer, Rebecca Lane, Melissa McKinnon, Iain Robertson

**Affiliations:** 1 Physiotherapy Department, Launceston General Hospital, Charles St, Launceston, Tasmania 7250 Australia; 2 Department of Physiotherapy, The University of Melbourne, Melbourne, Victoria 3052 Australia; 3 School of Primary Health Care, Faculty of Nursing, Medicine and Health Science, Monash University, Frankston, Victoria 3199 Australia; 4 Department of Physiotherapy, Princess Alexandra Hospital, Woolloongabba, Queensland 4102 Australia; 5 Physiotherapy Department, Northeast Health Wangaratta, Green Street, Wangaratta, Victoria 3677 Australia; 6 School of Physiotherapy, Faculty of Health Sciences, Australian Catholic University, Ballarat, Victoria 3350 Australia; 7 Biostatistician, Clifford Craig Foundation, Launceston General Hospital, Charles Street, Launceston, Tasmania 7250 Australia; 8 College of Health Sciences, University of Tasmania, Locked Bag 1320, Launceston, Tasmania 7250 Australia

**Keywords:** Emergency surgery, Abdominal surgery, Complications, Physiotherapy, Breathing exercises, Postoperative pulmonary complication, Pneumonia, Ileus, Rehabilitation, Patient education

## Abstract

**Background:**

Postoperative complications and delayed physical recovery are significant problems following emergency abdominal surgery. Physiotherapy aims to aid recovery and prevent complications in the acute phase after surgery and is commonplace in most first-world hospitals. Despite ubiquitous service provision, no well-designed, adequately powered, parallel-group, randomised controlled trial has investigated the effect of physiotherapy on the incidence of respiratory complications, paralytic ileus, rate of physical recovery, ongoing need for formal sub-acute rehabilitation, hospital length of stay, health-related quality of life, and mortality following emergency abdominal surgery. We hypothesise that an enhanced physiotherapy care package of additional education, breathing exercises, and early rehabilitation prevents postoperative complications and improves physical recovery following emergency abdominal surgery compared to standard care alone.

**Methods:**

The Incidence of Complications following Emergency Abdominal surgery: Get Exercising (ICEAGE) trial is a pragmatic, investigator-initiated, multicentre, patient- and assessor-blinded, parallel-group, active-placebo controlled randomised trial, powered for superiority. ICEAGE will compare standard care physiotherapy to an enhanced physiotherapy care package in 288 participants admitted for emergency abdominal surgery at three Australian hospitals. Participants will be randomised using concealed allocation to receive either standard care physiotherapy (education, single session of coached breathing exercises, and daily early ambulation for 15 min) or an enhanced physiotherapy care package (education, twice daily coached breathing exercises for a minimum 2 days, and 30 min of daily supervised early rehabilitation for minimum five postoperative days). The primary outcome is a respiratory complication within the first 14 postoperative hospital days assessed daily with standardised diagnostic criteria. Secondary outcomes include referral for sub-acute rehabilitation services, discharge destination, paralytic ileus, hospital length of stay and costs, intensive care unit utilisation, 90-day patient-reported complications and health-related quality of life and physical capacity, and mortality at 30 days and at 1 year following surgery.

**Discussion:**

The morbidity, mortality, and fiscal burdens following emergency abdominal surgery are some of the worst within surgery. Physiotherapy may be an effective, low-cost, minimal harm intervention to improve outcomes and reduce hospital utilisation following this surgery type. ICEAGE will test the benefits of this commonly provided intervention within a methodologically robust, multicentre, double-blinded, active-placebo controlled randomised trial.

**Trial registration:**

ACTRN 12615000318583. Registered 8 April 2015

**Electronic supplementary material:**

The online version of this article (10.1186/s13017-018-0189-y) contains supplementary material, which is available to authorized users.

## Background

Globally, millions of people every year require urgent time-critical emergency abdominal surgery to resolve potentially catastrophic small bowel obstructions, gastrointestinal tract perforations, haemorrhage, invasive cancerous tumours, blunt force/penetrative trauma injuries, and peritonitis [1]. Emergency surgery accounts for approximately 11% of total surgical cases in the USA yet disproportionately can contribute to half of all surgical deaths and a third of all complications [2]. Postoperative outcomes following emergency abdominal surgery are generally poorer when compared to elective surgery. The most common serious complication after emergency abdominal surgery is a postoperative pulmonary complication (PPC) with an incidence rate of 20–50% [3–5]. Emergency surgery is the single greatest risk factor for a PPC, much greater than the risk attributed to other types of surgery and existing patient comorbidities [5]. The consequences of developing a PPC are serious with doubled health costs [6], longer hospital stay [5, 7], and higher mortality [5, 7, 8]. Understandably, efforts to improve postoperative outcomes following emergency abdominal surgery are strongly advocated [9].

Promising findings of reduced morbidity and mortality following the introduction of multidisciplinary perioperative care bundles, including increased physiotherapy [10, 11], are yet to be confirmed in randomised controlled trials. The size of the contribution of each individual treatment component within a care bundle also limits interpretation of findings. It is unknown whether physiotherapy can independently improve outcomes after emergency abdominal surgery.

Although the provision of physiotherapy to emergency abdominal surgery patients is ubiquitous in high-income countries [12], no clinical trials exist that have investigated the effect of physiotherapy on PPCs and overall recovery in this high-risk, high-care burden cohort over and above standard care [13].

## Methods/design

### Trial objectives

The primary objective of the Incidence of Complications following Emergency Abdominal surgery: Get Exercising (ICEAGE) trial is to estimate the effectiveness of physiotherapy on the incidence of PPC, including pneumonia, in patients following emergency open abdominal surgery.

Secondary objectives are to estimate the possible effect physiotherapy may have on prolonged postoperative ileus incidence, rates of referrals for formal sub-acute rehabilitation services, discharge destination, hospital length of stay (LOS) and costs, readiness to discharge from hospital, intensive care unit (ICU) LOS and readmissions, and rate of change to physical function and quality of life (QOL) whilst in hospital and at 90 days, and 1-year mortality.

### Trial design

**Fig. 1:**
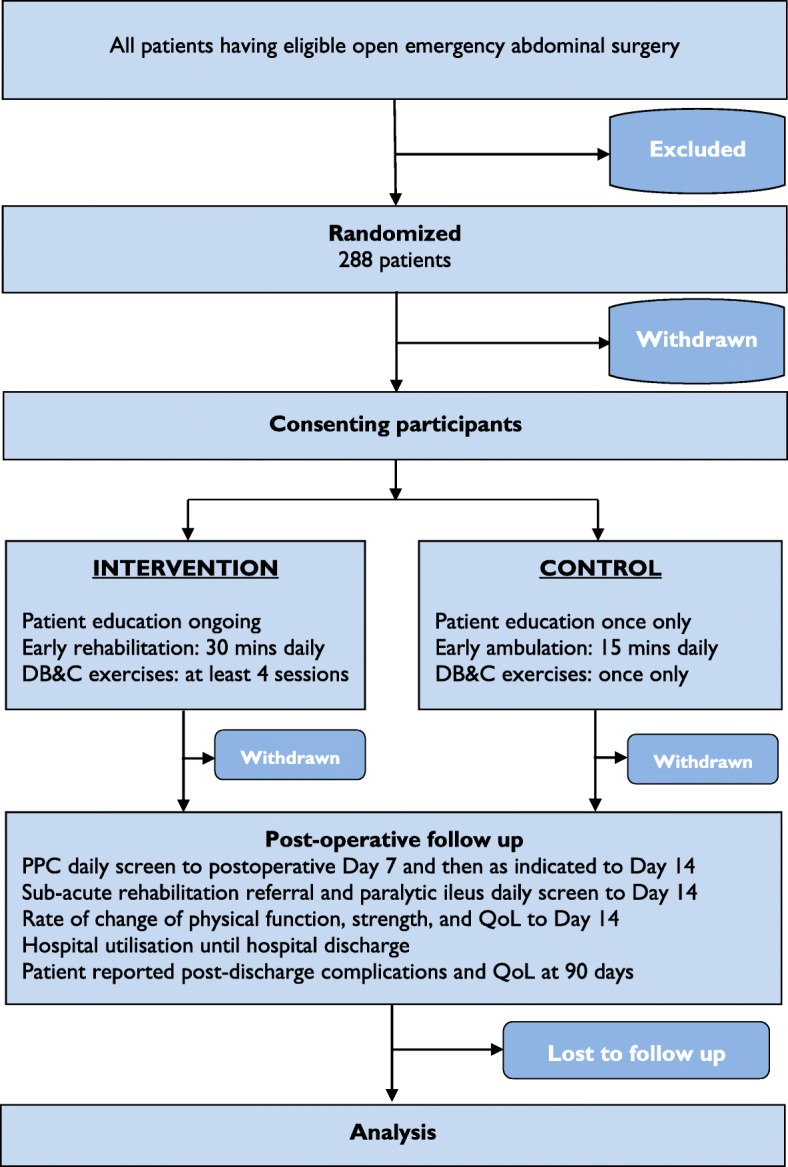
CONSORT diagram of the ICEAGE trial. Abbreviations: DB&C deep breathing and coughing, PPC postoperative pulmonary complications, HRQOL health-related quality of life

ICEAGE is a pragmatic, multicentre, parallel-group, randomised controlled trial. It is patient and assessor blinded, and powered for superiority. Eligible patients will be randomly assigned via concealed allocation into one of two physiotherapy treatment arms (Table 1). The control group will receive standard care physiotherapy from the first postoperative day consisting of education on prevention of complications with upright positioning, ambulation, and breathing exercises; a single deep breathing and coughing (DB&C) coaching session; and no more than 15 min of supervised ambulation once daily until a threshold score is met to discharge from this service. The intervention group will receive an enhanced physiotherapy care package from the first postoperative day consisting of (i) education on prevention of complications with upright positioning, ambulation, and breathing exercises; (ii) at least twice daily coached DB&C for the first two postoperative days; and (iii) at least 30 min of supervised early rehabilitation daily for the first five postoperative days. The mode of ambulation and respiratory physiotherapy will be standardised for both groups. The difference between groups will be (1) additional reminders and education as necessary for performance of independent exercises, (2) longer duration of ambulation and/or provision of individualised rehabilitation exercises, and (3) increased number of coached DB&C sessions (see Fig. 1 for the ICEAGE trial CONSORT diagram).

**Table 1:** TIDieR descriptions of ICEAGE interventions

TIDieR criterion	Control	Intervention
Item 1. Brief name: provide the name or a phrase that describes the intervention	Standard care: ‘talk, walk, breathe’	Enhanced physiotherapy care: ‘talk more, exercise more, breathe more’
Item 2 a) Describe any rationale, theory essential to the intervention	After emergency abdominal surgery the most common cause of morbidity and mortality is a postoperative pulmonary complication (PPC). Postoperative physiotherapy is routinely provided in intensive care units and surgical wards across Australia in an effort to prevent respiratory complications and enhance physical recovery after this major surgery type. Despite ubiquitous provision of this service, the effectiveness of postoperative physiotherapy is unknown.	Treatment components: ‘Talk more’: Repeated education sessions and reminders are allowed to be provided at the discretion of the physiotherapist. It is possible that more education will lead to greater motivation and increased independent performance of breathing and walking exercises.
Item 2 b) Describe goal of the elements essential to the intervention	Treatment components: ‘Talk’: The patient is informed of their risk of postoperative complications due to surgery and prolonged bed rest impairing their lungs and muscles. Education aims to inform, motivate, encourage, and inspire participation in exercises as prescribed. A booklet provides a written record of what was verbally taught. ‘Walk’: Early ambulation away from the bedside after surgery may prevent complications and physical decline after surgery. ‘Breathe’: Breathing and coughing exercises increase lung volumes, prevent postoperative atelectasis, improve gas exchange, clear stagnant secretions, and prevent postoperative pulmonary complications.	‘Exercise more’: The goal is to provide at least double the amount of physical activity daily. Daily physical activity will comprise of both ambulation and functional bed/chair/standing exercises. It is possible that there is a dose-dependent relationship with physical activity after surgery. Additional supervised physical activity early following surgery may hasten physical recovery and prevent complications more effectively. ‘Breathe more’: The goal is to provide at least four times the amount of coached breathing exercises in the first 2 days after surgery. It is possible that more breathing exercises performed early after surgery results in a greater risk reduction in the incidence of PPC.
Item 3. What (materials): describe any physical or informational materials used in the intervention, including those provided to participants or used in intervention delivery or in training of intervention providers.	Participant information materials: A booklet containing written and pictorial information regarding PPC and their potential prevention with early ambulation and breathing exercises to consolidate the learnt knowledge from verbal education and training. Intervention providers: Familiarisation with protocol prior to treating intervention participants. Badge cards containing the treatment protocol Template for documentation of treatment delivery Data entry sheets with further details of protocol	Participant information materials: As per control Intervention providers: As per control
Item 4. What (procedures): describe each of the procedures, activities, and/or processes used in the intervention, including any enabling or support activities	‘Talk’: once off Patients will have a single face-to-face education session following surgery with their physiotherapist. Patients will be educated on the benefit of walking and breathing exercises to prevent complications and improve recovery after surgery. Participants will be educated on the effect of surgery and bedrest to their lungs and muscles, and the benefit of walking and breathing exercises to prevent complications and improve recovery after surgery. Participants will be encouraged to perform the taught breathing exercises independently every hour during the day until they are walking at least 5 min, four times a day. Participants will also be strongly encouraged by their treating physiotherapist to sit upright, sit out of bed, walk away from their bedside (if safe to do so), or march on the spot beside their bed, as many times as they can during the following days to aid in the prevention of PPCs Participants will be provided with an education booklet. This booklet contains written and pictorial information about abdominal surgery, expected types of pain management, medical lines and drains, postoperative recovery process, and how to prevent postoperative respiratory complications with early ambulation and self-directed breathing exercises. The breathing exercises are prescribed within with the recommended prescription of reps and sets.	‘Talk more’: at least once, then as often as required As per the control group. In addition, intervention group participants will receive extra reminders and prompts during the first five postoperative days to perform the prescribed rehabilitation and breathing exercises independently. Pragmatically, the number of additional education sessions and the use of exercise diaries and instruction sheets will be at the discretion of each treating physiotherapist based on their clinical experience and the individual needs of the intervention group participant.
	‘Walk’: once daily ambulation only, < 15 min Initiation—as soon as practical after surgery Duration—no more than 15 min of total work time Frequency—once daily until a threshold score is met Intensity—rating of perceived exertion > 3/10, breathing deeper than at rest. Type—continuous activity for a minimum 1 min aiming for up to 15 min continuous as patient can tolerate. Allowable to train in intervals with work/rest ratio of 1:1 Mode—ambulation only. At every session the participant will be progressed sequentially through the protocol stages aiming to achieve a walking time of at least 10-min, but no more than 15-min. Successful ambulation is defined as continuously marching on the spot beside the bed or walking away from the bedside for more than 1 min. If a participant is unable to participate in upright ambulation, then no other non-ambulation exercises will be provided that day.	‘Exercise more’: once daily, ambulation and exercises, > 30 min Initiation—as soon as practical after surgery Duration—at least 30 min of total work time Frequency—once daily for at least the first 5 postoperative days and then until a threshold score is met Intensity—rating of perceived exertion > 3/10, breathing deeper than at rest. Type—continuous activity for a minimum 1 min aiming for as long as patient can tolerate. Allowable to train in intervals with work/rest ratio of 1:1 Mode—functional exercise programme starting with ambulation as per the control group protocol aiming for at least 15 min and if possible to at least 30 min of walking. If walking time is less than 30 min, non-ambulatory physical activity is continued to be provided until the minimum 30 min of activity time is achieved (not including rest periods). Physical activity is provided in a sequential step-down process starting with the highest activity possible and moving to less intense. These exercises consist of chair- or bed-based progressive low resistance, high repetition, lower and upper limb exercises of three–four sets as prescribed by the treating physiotherapist based on the individual patient’s requirements and functional ability. If a participant is unconscious or acutely unwell, functional electrical stimulation of muscles and/or passive movements of limbs can be used as a physical activity.
	‘Breathe’: once off coached session Participants will receive a single physiotherapy coached deep breathing and coughing (DB&C) session within the first 2 days following surgery, or once the ward physiotherapist considers a patient conscious enough to understand and participate in this session. Physiotherapists will treat patients in upright sitting either in a bed or in a chair. The coached DB&C exercises will consist of two sets of 10 slow-flow breaths to maximum inspiratory capacity with two to three inspiratory sniff breath-stacking manoeuvres at the end of each deep breath followed by a 3- to 5-s breath hold. Each set of 10 breaths are followed by three coughs, or a forced expiratory technique with an open glottis called a ‘huff’, with a small firm pillow, or suitable substitute, pressed over on the abdominal incision to support the wound and to encourage greater expiratory force. The treating physiotherapist will have the discretion to place hands on the patient’s chest wall during the coaching sessions to provide tactile feedback of performance. Total prescribed dosage of DB&C exercises is 20 breaths and six forced expiratory efforts every hour. The participants were encouraged to perform these every hour during the day until they were ambulant out of bed at least four times a day, for a minimum of 5 min at a time. No other prophylactic respiratory physiotherapy interventions or reminders to perform the taught DB&C exercises will be provided by the physiotherapist, this includes, but is not limited to, the following: manual or ventilator hyperinflation or recruitment manoeuvres in ventilated patients, manual techniques, positive expiratory pressure devices, incentive spirometers, or non-invasive ventilation.	‘Breathe more’: at least four coached sessions in the first 2 days: As per the control group. Additionally, intervention group participants will receive at least three additional physiotherapist-coached DB&C sessions in the first two postoperative days. At least four sessions will be provided in total. Beyond the second day additional, coached DB&C can be provided at the discretion of the treating physiotherapist if they consider the patient incapable of performing exercises unsupervised or they consider the patient requires additional respiratory exercises to prevent a PPC.
Item 5. Who provided: for each category of intervention, provider (for example, psychologist, nursing assistant), describe their expertise, background, and any specific training given	Qualified physiotherapists of varying experience levels at three different hospitals: new graduates, senior physiotherapists, and consultant level physiotherapists. An allied health assistant can deliver the ambulation protocol once this is deemed safe to do so by the treating physiotherapist.	Qualified physiotherapists of varying experience levels at three different hospitals: new graduates, senior physiotherapists, and consultant level physiotherapists. All sessions will be conducted by a physiotherapist (not an allied health assistant) until at least the fifth postoperative day unless the supervising physiotherapist is confident that an assistant or participant will undertake the rehabilitation programme exactly as prescribed for the minimum 30 min.
Item 6. How: describe the modes of delivery (such as face to face or by some other mechanism, such as internet or telephone) of the intervention and whether it was provided individually or in a group	Face-to-face, individual sessions	Face-to-face, individual sessions
Item 7. Where: describe the type(s) of location(s) where the intervention occurred, including any necessary infrastructure or relevant features	Inpatient hospital wards, patient bedsides at three government-funded, university affiliated, teaching hospitals Hospitals include a sub-regional secondary referral hospital, a regional primary referral hospital, and a metropolitan primary referral hospital.	Inpatient hospital wards, patient bedsides at three government-funded, university-affiliated, teaching hospitals. Hospitals include a sub-regional secondary referral hospital, a regional primary referral hospital, and a metropolitan primary referral hospital.
Item 8. When and how much: describe the number of times the intervention was delivered and over what period of time including the number of sessions, their schedule, and their duration, intensity or dose	See item 4	See item 4
Item 9. Tailoring: if the intervention was planned to be personalised, titrated or adapted, then describe what, why, when, and how	No tailoring	‘Talk’—Extra education sessions can be provided by physiotherapist as necessary based upon their clinical judgement and the requirements of the patient. ‘Exercise more’—Physiotherapist can incorporate specific functional exercises to target identified individual physical impairments, within the exercise protocol as long as the exercises comply with the goal intensity, duration, and type. ‘Breathe more’—Physiotherapist can provide more DB&C exercise sessions, in addition to the minimum of four coached sessions, in response to clinical judgement of risk and respiratory vulnerability.
Item 10. Modifications: If the intervention was modified during the course of the study, describe the changes (what, why, when, how)	N/A	N/A
Item 11. How well (planned): if intervention adherence or fidelity was assessed, describe how and by whom, and if any strategies were used to maintain or improve fidelity, describe them	Planned interim analysis of provision of ambulation programme to ensure that in > 80% of patients ambulation duration is no more than 15 min and provided until discharge criteria is met	Planned interim analysis of provision of exercise programme to ensure that in 80% of patients exercise duration is at least 30 min and provided for a minimum of five postoperative days/sessions.
Item 12: How well (actual): if intervention adherence or fidelity was assessed, describe the extent to which the intervention was delivered as planned	The trial will be amended or ceased for futility if there are critical failures to recruitment rates, breaches to protocol, or minimal treatment separation between groups.	The trial will be amended or ceased for futility if there are critical failures to recruitment rates, breaches to protocol, or minimal treatment separation between groups.

*Abbreviations: PPC* postoperative pulmonary complications, *DB&C* deep breathing and coughing

### Trial setting

The three participating centres represent a range of hospital types in Australia. The Launceston General Hospital (Launceston, Tasmania, Australia) is a regional primary referral hospital; the Princess Alexandra Hospital (Brisbane, Queensland, Australia), a metropolitan primary referral hospital; and Northeast Health Wangaratta (Wangaratta, Victoria, Australia), a sub-regional secondary referral hospital, are all government-funded, university-affiliated, teaching hospitals.

### Eligibility and exclusion criteria

Local investigators will screen daily surgery lists and recruit eligible patients from the ICU and wards at participating hospitals. Eligible patients will be adults over 18 years having open emergency abdominal surgery. This is defined as time-critical surgery (within 48 h of decision to operate) for a condition that is an immediate or intermediate threat to life or organ survival, involving at least one incision greater than, or equal to, 5 cm anywhere in the abdomen and through the fascia, for an existing hospital patient or a person admitted through the emergency department and requiring at least an overnight stay. Exclusion criteria are as follows: procedures solely involving inguinal hernia repairs, appendectomies, organ transplantation, thoracic, or gynaecological surgery; a PPC diagnosis anytime in the 24 h prior to eligibility screening; a pre-existing condition where the patient is unable to stand upright and ambulate for 1 min without a seated rest; severe cognitive impairment; unable to understand spoken English without the assistance of an interpreter (as determined by the research assistant, site investigator or ward physiotherapist performing eligibility screening); within 7 days of elective abdominal surgery; planned repeat surgery within 5 days of emergency procedure; approaching imminent death or withdrawal of medical treatment within 48 h of their surgical procedure; medical orders not to participate in early active rehabilitation; patients unable to be randomised by the research team within 48 h of surgery; enrolment in another clinical trial with similar endpoints; and previous enrolment in ICEAGE.

### Randomisation and allocation

An administrative assistant independent of the trial will prepare 288 consecutively numbered (1 to 288) opaque envelopes each containing an allocation card wrapped in extra paper or aluminium foil [14]. Allocation sequence is determined by a computer-generated (http://www.randomizer.org/) blocked random number table (6 blocks of 48) with large blocks to minimise guessing of allocation sequence. The randomisation table will be sealed in an opaque envelope, locked within the primary centres’ research institute, and made unavailable to trial personnel until trial completion. The number of consecutively numbered envelopes provided to each site will be dependent on funding agreements (i.e. funded to recruit one block of 48 participants, or, on a per patient recruit basis till the end of the trial).

Once a patient has been deemed eligible, the treating ward physiotherapist receives the group allocation by first writing patient details on the next consecutively numbered sealed opaque envelope and then opening it. If numerous new patients are eligible on the same day at a site, envelopes will be allocated in alphabetical order of surname. If an envelope is not available at the time of randomisation, a coin can be tossed by the treating physiotherapist and the result written on a card and countersigned by an independent observer. This card will be placed within the envelope that should have been allocated for that patient. If an envelope is erroneously opened out of sequence, the reason for doing so will be documented on it and the patient will be treated as per allocation card. Any aberrations to sequential allocation will be recorded. Envelopes will be kept securely by the ward physiotherapist or site investigator until hospital discharge and then integrated with the consent form and filed securely by the site investigator. Potential selection bias will be studied by extracting basic demographic data and planned surgical procedure from the medical records of all included and excluded patients.

### Trial withdrawal

A patient is withdrawn if they withdraw their consent or do not provide consent following randomisation.

### Trial interventions

#### Standard care physiotherapy (control)

The control group will receive physiotherapy care that is typically delivered in Australian hospitals^12^. This comprises of education (talk), early supervised ambulation (walk) once daily from the first postoperative day, and a single session of coached DB&C exercises (breathe) (Table 1).

##### Patient education—‘talk’

Patients will be educated on their risk of a PPC and slow recovery after surgery. They will be educated on the benefit of walking and breathing exercises to prevent complications and improve recovery after surgery [15, 16]. Following the first successful ambulation session, all participants will be strongly encouraged by their treating physiotherapist to sit upright, sit out of bed, walk away from their bedside (if safe to do so), or march on the spot beside their bed, as many times as they can during the following days to aid in the prevention of PPCs. They will be encouraged to seek assistance from a nurse if necessary and to walk with their visitors, or independently, once they are able to walk away from the bedside safely. Patients will be encouraged to perform the coached DB&C exercises independently every hour during the day until they are walking at least 5 min, four times a day. Patients will be provided with an information booklet containing all the supplied verbal information in written and pictorial format.

##### Early supervised ambulation—‘walk’

The standardised ambulation protocol [16, 17] (Table 2) will be provided as soon as practical after surgery by a physiotherapist. Patients will receive one supervised ambulation session per day until a threshold score is met [18] (Additional file 1), or discharge from hospital, whichever occurs first. At every session, the participant will be progressed sequentially through the protocol stages aiming to achieve a walking time of at least 10 min, but no more than 15 min, at an intensity of at least three on the Borg 10-point visual analogue scale (VAS) of perceived exertion [19], and where breathing is deeper than at rest. The treating physiotherapist will use a stopwatch to ensure the treatment session does not exceed 15 min and can provide a walking aid if clinically indicated. If necessary, ambulation sessions can comprise of equal work/rest intervals. Shorter, but not longer, rest times are allowable. The final achieved ambulation stage corresponds to total time walked, including the marching on the spot time, and not including rest periods.

**Table 2:** ICEAGE ambulation protocol

Stage 1 (safety)	Sit over edge of bed/sit in chair minimum of 2 min
Stage 2 (safety)	March on spot 0–1 min
Stage 3 (ambulation)	March on spot/walk away from bedside 1–3 min
Stage 4 (ambulation)	March on spot/walk away from bedside 3–6 min
Stage 5 (ambulation)	Walk away from bedside 6–10 min
Stage 6 (ambulation)	Walk away from bedside 10–15 min
Stage 7 (ambulation)	Walk away from bedside > 15 min
Provide assisted early ambulation as soon as possible on the first postoperative day. At each session, progress through each stage in sequence. Time achieved in the session is accumulative. Aim to achieve rating of perceived exertion of greater than 3/10. Aim to assist patient to ambulate more than 10 min (stage 6 or greater). Once patient is able to ambulate past stage 3, patient can be assisted to ambulate with a physiotherapy assistant, as long as safe to do as determined by the ward physiotherapist. Interval training is permissible to obtain target walking time. Each interval of rest time must not exceed the preceding work time. Total session time is the accumulative work time. Provide assisted early ambulation once a day until discharged according to the discharge scoring tool.	

Successful ambulation is defined as continuously marching on the spot beside the bed or walking away from the bedside for more than 1 min. Time from surgery to time of first successful ambulation will be recorded. If participants are unavailable or unable to achieve ambulation for more than 1 min, the assisted ambulation session will be attempted once again on the same day. The reasons for participants being unable to ambulate or not achieving a minimum of 10 min will be recorded. If a participant is unable to participate in upright ambulation, then no other non-ambulatory exercises will be provided that day. All treating therapists will be provided with protocol badge cards and trained by the site investigator. An allied health assistant can deliver the ambulation protocol once this is deemed safe to do so by the treating physiotherapist. The professional qualification of the person assisting each ambulation session will be documented.

##### Coached respiratory exercises—‘breathe’

Participants will receive a single physiotherapy education and coached DB&C session within the first 2 days following surgery, or once the ward physiotherapist considers a patient cognitively capable of participation.

Physiotherapists will treat patients in upright sitting either in a bed or in a chair. The coached DB&C exercises will consist of two sets of 10 slow-flow breaths to maximum inspiratory capacity with two to three inspiratory sniff breath-stacking manoeuvres at the end of each deep breath followed by a 3- to 5-s breath hold [16, 17]. Each set of 10 breaths are followed by three coughs or forced expiratory technique with an open glottis called a ‘huff’, with a small firm pillow, or suitable substitute, pressed over on the abdominal incision to support the wound and encourage greater expiratory force. The treating physiotherapist will have the discretion to place hands on the patient’s chest wall during the coaching sessions to provide tactile feedback of performance. Total prescribed dosage of DB&C exercises is 20 breaths and six forced expiratory efforts every hour. No other prophylactic respiratory physiotherapy interventions or reminders to perform the taught DB&C exercises will be provided by the physiotherapist; this includes, but is not limited to, the following: manual or ventilator hyperinflation or recruitment manoeuvres in ventilated patients, manual techniques, positive expiratory pressure devices, or non-invasive ventilation.

Of note, all participants at Princess Alexandra Hospital and Northeast Health Wangaratta will be provided with an incentive spirometer by the nursing staff in the first 2 days after surgery as per routine nursing care at these hospitals. According to the pragmatic nature of this trial, this practice will continue and the incentive spirometer use will be recorded. Physiotherapists will not encourage incentive spirometer use in the control group.

#### Enhanced physiotherapy care (intervention)

The intervention group will receive the same care as the control group with the addition of the following (Table 1):

##### Patient education—‘talk more’

Education and an information booklet will be provided as per the control group [15, 16]. In addition, intervention group participants will receive extra reminders and prompts during the first five postoperative days to perform the prescribed rehabilitation and breathing exercises independently. Pragmatically, the number of additional education sessions and the use of exercise diaries and instruction sheets will be at the discretion of each treating physiotherapist based on their clinical experience and the individual needs of the participant.

##### Supervised early rehabilitation—‘exercise more’

**Fig. 2:**
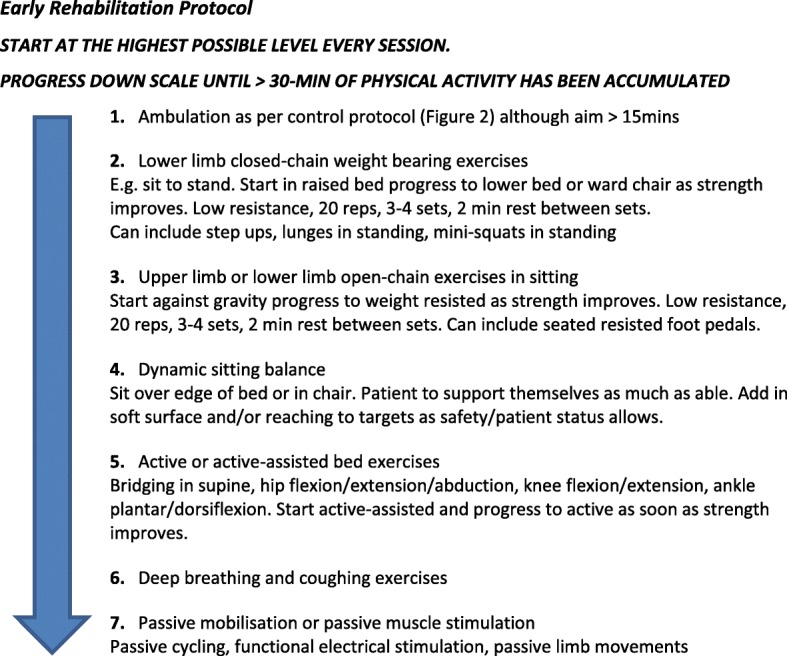
ICEAGE exercise protocol. reps repetitions

Intervention participants will receive physiotherapist supervised early rehabilitation lasting at least 30 min provided at least once daily. Rehabilitation will consist of a combination of ambulation and non-ambulatory chair- or bed-based exercises (Fig. 2) starting as soon as possible after surgery and continuing daily thereafter for at least the next five postoperative days or until at least five daily sessions are delivered. Exercises will be provided in a top-down hierarchical sequence starting from ambulation and aiming for the maximum physical activity type the participant is able to attempt. Exercise delivery progresses sequentially down the protocol until at least 30 min of activity have been accumulated. For example, participants will first be assisted to ambulate for at least 15 min with a goal to achieve at least 30 min. If time ambulated is less than 30 min, the participant will attempt resistive lower limb exercises in standing for the remaining time or until fatigue limits the performance. If time still remains less than 30 min, participants will attempt upper limb/lower limb resistance exercises in sitting until fatigued. This process continues until a minimum total treatment time of 30 min has been reached. A treating physiotherapist is also permitted to prescribe a personalised training activity based on a detected functional deficit to be incorporated within the rehabilitation programme. These targeted and individualised sessions will be recorded.

This early rehabilitation protocol has been designed so that critically unwell ICU patients can participate [20, 21]. A conscious, intubated, and ventilated patient could be assisted out of bed to march on the spot once assessed as safe [20, 21], and an unconscious patient could participate in 30 min of passive limb movement [22, 23] and/or functional electrical stimulation of major muscles [24]. The initial starting exercise for each rehabilitation session will be made pragmatically at the discretion of the treating physiotherapist using their own past experience and knowledge base regarding the participant’s current clinical and physiological status. No specific sedation score or assessment screening tool will be used to guide this decision.

Supervised activity in the intervention exercise protocol must aim to be continuous for more than 1 min, at an intensity of at least three on the Borg 10-point VAS of perceived exertion [18] and where breathing is deeper than at rest. The exception to this is for the passive limb exercises and functional electrical stimulation of muscles provided to unconscious or acutely unwell participants. Interval training is permitted; however, rest time cannot exceed the preceding active period. Once rest time becomes greater than the preceding work time, the exercise session is considered completed. The treating physiotherapist will use a stopwatch to ensure each rehabilitation session equals or exceeds 30 min. If a participant is unable to complete a 30-min session, the treating physiotherapist will document the reason. A further rehabilitation session later in the same day may be attempted to reach the 30-min threshold.

All sessions will be conducted by a physiotherapist until at least the fifth postoperative day unless the supervising physiotherapist is confident that a therapy assistant or the participant will undertake the rehabilitation programme exactly as prescribed for the minimum 30 min. After the fifth postoperative day, daily treatment with a physiotherapist or therapy assistant will continue until either a referral for sub-acute rehabilitation services is made (see endpoints), a participant is discharged from hospital, or once a threshold score is met [18] (Additional file 1), whichever occurs first. If the patient meets the discharge score prior to the fifth postoperative day, daily treatment will continue until the fifth postoperative day but cease thereafter.

##### Coached respiratory exercises—‘breathe more’

The mode, type, and timing of delivery of DB&C exercises will be provided as per the control group. Intervention group participants will receive at least three additional physiotherapist-coached DB&C sessions in the first two postoperative days. At least four sessions will be provided in total. The total number of sessions will be recorded. Beyond the second day, additional coached DB&C can be provided at the discretion of the treating physiotherapist if they consider the patient incapable of performing exercises unsupervised or requires additional respiratory exercises to prevent a PPC are indicated. Similar to the control group, no additional prophylactic respiratory interventions apart from the DB&C exercises will be provided.

#### Other components of postoperative care

Additional ambulation occasions or DB&C sessions conducted independently by the participant or assisted by other ward staff will not be measured nor controlled for as this would not be feasible or consistent with the pragmatic nature of this trial. All other aspects of patient care, including general anaesthesia, surgical techniques, intraoperative ventilation parameters, fluid delivery, prophylactic antibiotic prescription, pain management, use of lines and drains, general nursing care, and discharge planning, will be administered according to routine clinical practice at each participating centre.

The three participating sites have limited physiotherapy service on weekends and on public holidays. If protocol is unable to be delivered due to service limitations, this will be recorded.

#### Safety monitoring

Early rehabilitation of at least 30 min daily in ICU patents is safe when provided in consideration of the patient’s immediate clinical status and haemodynamic stability [25]. The emergency abdominal surgery population, in comparison, will be generally less acute. No adverse events in this population have been reported in similar exercise treatment protocols [26]. The following events will be reported if they occur during a treatment session and only if it is standard procedure at each site to monitor these parameters during physiotherapy treatment: symptomatic blood pressure or heart rate change 20% from resting, new cardiac arrhythmia, or drop in oxygen saturation > 10% from baseline, which fails to recover within 2 min of ceasing activity; patient requires increased sedation or inotropic support; line detachment; fall; or severe nausea. Adverse events will be immediately recorded in the participant’s medical record and in the case report form. The treating physiotherapist will immediately report the event to the site investigator who will alert the chief investigator within 24 h of the event for further investigation and classification. A significant adverse event (SAE) occurs where, in the opinion of the chief investigator, the event was directly related to the intervention or control treatments, was unexpected, and significantly impacted the participant’s immediate health and/or delayed recovery. All SAE will be reported to the ethics committee.

### Outcome measures

#### Primary outcome

The primary outcome is the first occurrence of a PPC within the first 14 postoperative hospital days. PPCs will be defined by the Melbourne Group Score (Table 3) which is reliable, valid [27, 28], sensitive to interventions [16], and has high inter-rater reliability [29]. It has eight clinical criteria: four factors relating to symptoms and four to diagnostic markers. A PPC will be diagnosed when four or more factors are present at any time from midnight to midnight on one postoperative day. The day a PPC is first diagnosed is the day of onset for purposes of time-to-event analysis.

**Table 3:** PPC diagnostic criteria—Melbourne Group Score Version 3

When four or more of the following criteria^*^ are present anytime in the 24-h period 00:01 to 24:00 on a single postoperative day: 1. New abnormal breath sounds on auscultation different to preoperative assessment^+^ 2. Production of yellow, green, or brown sputum different to pre-morbid status^+^ 3. Pulse oximetry oxygen saturation (Sp0_2_) < 90% on room air or FiO2 on 0.21 on more than one consecutive postoperative day^**^ 4. Raised maximum tympanic temperature ≥ 38 °C on more than one consecutive postoperative day 5. Chest radiograph report of collapse/consolidation^# ***^ 6. Presence of infective organisms on sputum culture report^***^ 7. White cell count > 11 or < 3 8. Physician’s diagnosis of postoperative pulmonary complication (e.g. atelectasis, pneumonia, AECOPD, respiratory failure, upper respiratory tract infection) OR prescription of an antibiotic specific for respiratory infection	

*Abbreviations*: *PPC* postoperative pulmonary complications, *SpO2* pulse oximetry, *FiO2* fraction of inspired oxygen, *AECOPD* acute exacerbation of chronic obstructive pulmonary disease, *PEEP* positive end expiratory pressure, *CXR* chest X-ray

*If a blinded physiotherapist, nurse, or physician documents in the medical record the occurrence of a criterion, it can be taken as a positive finding. If no documentation present, a blinded assessor is required to assess this directly

^+^If no preoperative assessment or documentation assume normal at baseline

**For ventilated patients, if FiO_2_ ≥ 0.5 or PEEP ≥ 8, assume criterion 3 is present (do not alter FiO_2_), for all other patients set FiO_2_ to 0.21 and observe SpO_2_ for 2 min. If SpO_2_ drops below 90%, immediately reinstate previous FiO_2_. If not permissible to alter ventilation parameters, assume positive

^#^If no written report for a CXR is available and a patient has three other positive signs, a masked senior physiotherapist or ward medical officer is to be contacted to report verbally on the available CXR

***When there are no daily measures of CXR or sputum sampling, carry over a positive finding to the next consecutive postoperative day

Participants are assessed prospectively and daily for a PPC by a blinded assessor until the seventh postoperative day. From the 8th to the 14th postoperative day, additional PPC assessments are performed only as clinically suspected based on signs or symptoms of respiratory system deterioration reported within the medical record. To reduce the potential for missing data, retrospective collection of PPC data from the medical record will be permitted when a patient or assessor is unavailable for PPC assessment. Diagnostic components (chest X-ray (CXR), white cell count, sputum microbiology) are recorded only if results are available. All medical officers are masked to group allocation, and these diagnostic tests are ordered only as clinically indicated, and not routinely for the purposes of the ICEAGE trial.

For this trial, modifications have been made to reduce delays to possible diagnosis of a PPC (Table 3). An automatic positive threshold for the hypoxemia criterion has been set for ventilated patients; a positive CXR and/or sputum sample finding can be carried over to the next postoperative day if serial testing was not performed; a CXR can be verbally reported by a blinded senior respiratory physiotherapist or physician, rather than awaiting a radiologist report; and when three factors (out of a possible eight) are present, the blinded site investigator or ward physiotherapist will discuss the option of further diagnostic testing to confirm PPC with the participant’s treating physician. Additionally, patients with three out of eight criteria will be assessed twice daily for any clinical deterioration.

Any suspected PPC will be confirmed by a blinded senior physiotherapist, and the participant will then receive respiratory treatment as determined by the ward physiotherapist.

#### Secondary/exploratory trial outcomes

In hierarchical order:

1. Referral by the surgical team or other allied health professional within the first 14 postoperative days for additional acute-ward physiotherapy rehabilitation, sub-acute hospital rehabilitation, or community-based rehabilitation. On receipt of a referral, or by the 14th postoperative day if referral not received, a blinded senior physiotherapist will review the participant’s current medical record and adjudicate for the requirement for rehabilitation services.
2. Discharge destination from the acute hospital ward (home, rehabilitation facility, nursing home, or other hospital);
3. Hospital LOS in days. This is defined as continuous days spent in any type of inpatient hospital service (i.e. acute care, sub-acute rehabilitation, or time at another hospital) from the day of admission for acute condition to the day of discharge to community. Hospital days will be sub-categorised into acute and sub-acute;
4. Incidence of a prolonged postoperative ileus (PPOI) anytime from the fourth to the 14th postoperative hospital day. A PPOI is defined if two or more of the following criteria are met on or after the fourth postoperative day [30]: (i) nausea or vomiting, (ii) inability to tolerate an oral diet over last 24 h, (iii) absence of flatus over the last 24 h, (iv) abdominal distension, and (v) radiologic confirmation. The day which PPOI is first diagnosed is the day of PPOI onset for purposes of time-to-event analysis.
5. Diagnosis of pneumonia defined as the presence of new CXR infiltrates along with at least two of the following: temperature > 38 °C, dyspnoea, cough, and purulent sputum, altered respiratory auscultation, and white cell count > 14,000/ml or leukopenia < 3000/ml on any day within the first 14 postoperative hospital days;
6. Recovery of physical function and QOL
  i) Rate of change of the Modified Iowa Level of Assistance [31] (mILOA; Additional file 1) score over four measures taken second daily from the first postoperative day and a final score on the day of acute-ward discharge. The mILOA consists of four mobility tasks (supine to sitting on the edge of the bed, sit to stand, walking, and negotiation of one step), which are graded according to the level of assistance required, use of gait aid, and the distance that can be walked. Data will be extracted from the medical and physiotherapy records to score;
  ii) Rate of change in handgrip strength (kg) measured using a handheld dynamometer over four postoperative measures within the first 14 days. Measures will be taken at least 2 days apart;
  iii) Rate in change of the EuroQual QOL score [32] (EQ-5D; Additional file 2); measures to be taken directly from the patient over two consecutive measures separated by 48 h, within the first five postoperative days, and at 90 days following surgery;
  iv) Health-related QOL using World Health Organisation Disability Assessment Schedule V2 [33] (WHODAS; Additional file 3) change from self-rated status in the week prior to surgery, to day of discharge from acute care, and at 90 days following surgery;
  v) Physical capacity using Self-Assessment of Physical Activity Questionnaire [34] (SAQ; Additional file 4) from self-rated capacity regarding the week prior to surgery to 90 days following surgery;
  vi) Time in hours from end of operation to time able to achieve ambulation greater than 1 min;
  vii) Time in days from end of operation to achieve ambulation greater than 10 min;
7. ICU LOS in days;
8. Unplanned ICU admission during acute hospital stay;
9. Mortality—in-hospital, 30 and 90days, and at 1 year;
10. Sepsis within the first 7 days according to the Sequential Organ Failure Assessment (SOFA) for ICU patients and quick-SOFA for ward patients [35];
11. Time in days to discharge from physiotherapy as determined daily using the Post-Operative Physiotherapy Discharge Scoring Tool [18] (Additional file 1);
12. Time in days to readiness for hospital discharge within the first 14 postoperative days defined by standardised scoring criteria [36];
13. Patient reported complications at 90 days using a standardised semi-structured interview [16];
14. Hospital costs for the admission episode of care supplied by the participating centres’, or health departments’, costing data for each participant’s admission episode.

**Fig. 3:**
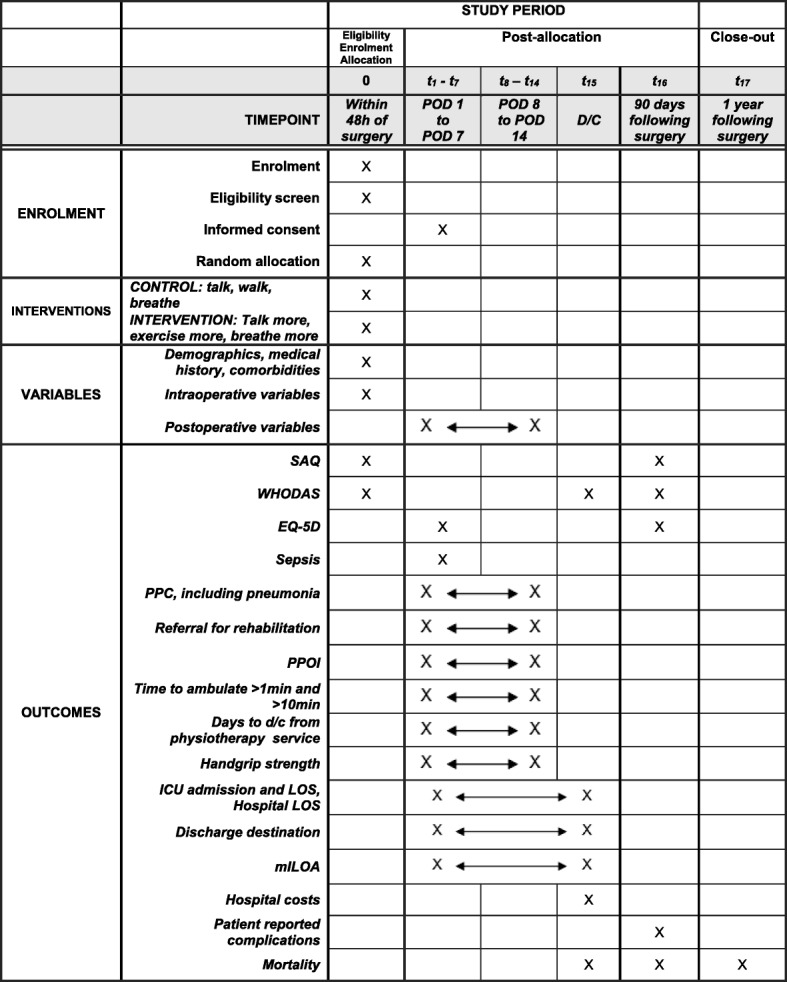
ICEAGE schedule of events and timeline. Abbreviations: POD postoperative day, D/C hospital discharge, SAQ specific activity questionnaire, WHODAS World Health Organisation disability assessment score, EQ-5D EuroQuol five domains, PPC postoperative pulmonary complication, PPOI prolonged postoperative ileus, ICU intensive care unit, LOS length of stay, mILOA modified Iowa level of assistance

WHODAS, EQ-5D, and SAQ measures will be via direct interview whilst in hospital and by phone interview when patient at home. At 90-day (± 14 days) follow-up, if participant is unable to be contacted for a period of five consecutive working days, a standardised cover letter, questionnaires, and self-addressed return paid envelope will be posted. Forms not returned within 2 weeks will be considered lost to follow-up for post-discharge secondary outcomes. Figure 3 describes the schedule of events, trial conduct, and outcome measurements.

### Protocol endpoints

Treatment protocols will be ceased when:

1. Participant is discharged or transferred from the primary treating hospital.
2. Participant becomes deceased or death is expected within next 7 days.
3. Participants meet a threshold score to trigger discharge from physiotherapy treatment^18^. This criterion is delayed for the first five postoperative days for intervention participants.
4. A confirmed PPC diagnosis ceases the respiratory treatment protocol. Respiratory treatment is then provided at the discretion of the ward physiotherapist. Participants remain being treated with the rehabilitation protocol as per group allocation.
5. A confirmed referral for additional sub-acute rehabilitation services ceases the ambulation or early rehabilitation protocol. Rehabilitation is then provided at the discretion of the ward physiotherapist. Participants remain being treated with the respiratory treatment protocol as per group allocation.

### Blinding

The recruiting and treating physiotherapists will be solely aware of group allocation. Assessments of primary and secondary outcomes and data collection will be performed by independent masked assessors not involved in the participant’s care. Assessors will require access to the medical record. To ensure masking to group allocation, documentation of physiotherapy assessments and interventions will be written to a standardised format (Additional file 5) with treatment recorded ‘Treatment provided as per ICEAGE protocol’. Documentation detailing physiotherapy treatment will be recorded separately and stored securely by the treating physiotherapist until the 14th postoperative day, the day of discharge, or until a defined endpoint is met, whichever occurs first, at which point the physiotherapy treatment documentation will be integrated into the medical record.

All doctors, surgeons, nurses, discharge coordinators, data analysts, and statisticians will be blinded to group allocation. If a participant inadvertently informs an assessor of their specific physiotherapy treatment, this will be reported, and a new assessor will be found for ongoing assessments.

It is anticipated that participants will be blinded to group allocations as both arms receive physiotherapy. A convenience sample of 30 consecutive participants will be asked which group they believed they had been allocated at the 90-day follow-up interview.

### Data management

The data dictionary for ICEAGE is supplied in Additional file 6. Data will be collected from participants using a standardised electronic case report form (CRF) and stored in participating centres’ password protected electronic hard drives. To ensure correct data entry, the CRF has been designed with extensive use of data entry limitation rules and on-screen prompts. Site investigators will be required to perform random covert audits of data collected by trial personnel during the trial for reliability and correctness against the medical record. There are no industrial contractual arrangements in relation to the de-identified data. On completion of the trial, the database will be made available for independent analysis or as an appendix in the publishing journal if requested.

### Statistical analysis

#### Sample size

PPC rates previously reported following emergency abdominal surgery are between 30 and 40% [3, 4, 27]. A 2013 prospective audit (unpublished) of PPC incidence in 50 consecutive patients at the primary participating centre found a rate of 20% (95%CI 10–34%). A 60% relative risk reduction in PPC with timely physiotherapy can be achieved in the elective abdominal surgery population [16]. A total sample size of 262 completed participants (131 per group) is required to detect a reduction in PPC rate by 60% from an anticipated baseline of 20% (20% down to 8%) with an alpha 0.05 and power 80%. This is arbitrarily increased to compensate for a possible 10% drop out/withdrawal rate and uncertainty around baseline incidence and effect size. In total, a sample of 288 participants is required for this trial.

#### Statistical methods

Imbalances of potential confounding variables between trial groups at recruitment will be assessed. Adjustment covariates will be selected by backward stepwise regression from covariates that are anticipated to affect incidence of PPC. These include respiratory comorbidity, smoking history, age, gender, length of operation, operation category (upper gastrointestinal, colorectal, urological, other), and ICU admission immediately following the procedure.

##### Primary outcome

The absolute and relative rates of PPC in the trial groups will be estimated using multivariate robust random effects Poisson generalised linear regression to allow assessment of binary outcomes with or without adjustment for potential confounding variables (incidence rates and rate ratios, 95% confidence intervals, *p* values). Treatment centre will be treated as a fixed variable in multi-level models. In addition, the effect of time from the end of surgery/anaesthesia to commencement of symptoms of PPC will be compared using Cox proportional hazards regression with and without covariate adjustment (hazards ratio, 95% confidence intervals, *p* values). A Kaplan-Meier graphic representation of this analysis will be performed.

##### Secondary outcomes

1. Binomial outcomes including rehabilitation referrals, pneumonia, PPOI, unplanned ICU admissions, patient-reported complications, and mortality will be analysed using multivariate robust random effects Poisson generalised linear regression.
2. Time-to-event binomial outcomes (e.g. PPC, pneumonia, PPOI, achievement of ambulation goals of 1 and 10 min; and hospital and ICU LOS; and mortality) will be compared by estimating hazard ratios using Cox proportional hazards regression, with and without covariate adjustment where necessary and graphically illustrated using Kaplan-Meier methods.
3. Measures involving estimation of rate of change over time (QOL and functional capacity) will be analysed using repeated-measures mixed effects linear regression or ordered logistic regression for rank-ordered scale measures.
4. Hospital costs will be compared using mixed effects linear regression. Log transformation of highly skewed cost data will be performed. Depending on the adequacy of the data for purposes of economic analysis, a cost-utility may be conducted.

All outcomes will be analysed on an intention-to-treat basis. An intention-to-protocol sensitivity analysis will be performed by identifying as a separate group participants who received more than 80% of planned protocol sessions as per group allocation. Other planned sensitivity analyses will be made according to age (> 65), surgery type (colorectal, upper gastrointestinal, other), preoperative self-reported physical capacity, and gender. Sensitivity of outcome estimates to missing data will be evaluated using multiple imputations. All analyses will be performed using Stata MP2 V14 (StataCorp, College Station, TX, USA).

#### Trial monitoring

The steering committee consists of the chief investigator, site investigators, and an academic investigator who contributed to the design and revision of the study protocol. The chief investigator is responsible for the study administrative management, communication with local investigators, and assists participating centres with trial conduct, record keeping, and data management. A planned interim analysis 1 year into trial, or at *n* = 50, whichever occurs first, will consider trial conduct, recruitment, adverse events, and adherence to trial protocol. The volume and duration of provided physiotherapy will be compared between groups. The trial will be amended or ceased due to futility if there are critical failures to recruitment rates, breaches to protocol, or minimal treatment separation between groups. Primary or secondary outcome differences will not be investigated at this interim analysis as this could lead to a subconscious bias in trial conduct.

### Ethics and dissemination

#### Retrospective consent

Ethics approval has been granted for eligible patients to be randomised prior to consent. Patients within the first two postoperative days after emergency abdominal surgery are often unwell and in an emotionally vulnerable state. Initial ethics approval was granted with a traditional enrolment into the trial following gaining of consent. However, following a planned interim analysis of trial conduct on the 50th patient recruited into the trial (unpublished data), patients and next of kin reported feeling distressed when approached by researchers in the first two postoperative days to discuss the trial. Further detailed feedback on the consenting process was sought from patients (*n* = 20) at 1-week post-surgery. All patients reported feeling too unwell in the first few days after surgery and disliked having to consider participating in a trial that was comparing two forms of low-risk commonly provided care. All patients indicated that they would prefer to provide retrospective consent. They recommended approximately no earlier than the third postoperative day or when they were not feeling nauseated. The two comparison interventions had nil documented occasions of harm at that point in the trial. Adverse events are similarly very low (< 1%) in critically unwell patients who are provided with 30 min of daily rehabilitation therapy [25]. There is minimal clinical risk to the patient being provided with either two arms of the study. Randomisation immediately on eligibility also allows the physiotherapy protocol to be delivered without delay, possibly enhancing the clinical outcome for all participants. Additionally, the primary outcome measure is non-invasive, has minimal risk, and requires no additional participation from the patient outside of standard care. An application to all responsible ethics committees was placed to alter consent to a retrospective consent following randomisation and was granted.

The principal site investigator will approach the patient from the fourth postoperative day and describe the trial, including the potential risks and benefits, and involvement required from the patient. Written information will be provided, and the patient will be invited to participate in the trial. Any person not consenting following randomisation is withdrawn from the trial. If the patient consents to continue in the trial, they will sign a consent form as required by local ethics committees and in accordance with the Declaration of Helsinki. If the patient remains in a state where informed consent is unable to be provided, (i.e. unconscious, delirious, nauseated, pain, repeat surgery) the next of kin will be contacted for consent. If the patient or next of kin declines to participate, the patient will take no further part in the trial and all data will be withdrawn. Physiotherapy will then be provided to them at the discretion of the ward physiotherapist.

Trial participants will have the autonomy to decline any therapy session as per standard care provision and medico-legal requirements regardless of recruitment into the trial. Reasons for non-participation in therapy sessions will be documented.

#### Dissemination

This protocol is reported according to the SPIRIT guideline [37]. Trial results will be written in accordance with the CONSORT extensions for a pragmatic trial using a non-pharmacological intervention, published in a peer-reviewed journal, and presented at national and international meetings.

#### Trial status

This trial is currently active. First participant was recruited on 25 June 2015. It is anticipated that recruitment will be completed in late 2018.

## Discussion

ICEAGE will provide the first high-level degree of evidence for the possible benefit of physiotherapy to reduce complications and improve physical recovery following emergency abdominal surgery. This trial involves three different types of hospitals: rural, regional, and metropolitan; includes most types of open major emergency abdominal surgery; and is conducted within a pragmatic framework where all other perioperative care is conducted without change. Our findings will be highly generalizable to most first-world hospitals.

In keeping with normal clinical practice and pragmatic trial methodology, it is unlikely that the interventions will be provided exactly as per protocol for every planned session; indeed, there will be many occasions where it will not be possible to treat a patient as planned due to extraneous circumstances (e.g. away at imaging, returned to theatre, refuses treatment, sudden deterioration). To ensure that our findings are relevant to practical real-life clinical situations in which our interventions are intended to be applied in, all patients entered in the trial will be analysed according to intention-to-treat principles, regardless of adherence to protocol. To determine if results are affected by adherence, we have planned a per-protocol sensitivity analysis of those patients who adhered to the protocol in more than 80% of intended occasions.

All assessors and patients will be masked to group allocation, and there is an active-placebo of current practice physiotherapy. This will ensure that any effects are independently associated with the additional therapy provided rather than the Hawthorne effect of health professional interaction. ICEAGE will include not only short-term clinical outcomes, but medium-term patient-reported QOL and physical recovery, and long-term mortality. The results of ICEAGE will inform the clinical provision of physiotherapy to millions of patients globally every year.

## Additional files

Additional file 1: Postoperative Physiotherapy Discharge Scoring Tool. Table describing the discharge from physiotherapy scoring. (DOCX 15 kb)

Additional file 2: EQ-5D-5L. EuroQual 5 Domains scoring. (DOCX 90 kb)

Additional file 3: WHODAS. World Health Organisation disability assessment scale. (DOCX 20 kb)

Additional file 4: WHODAS. World Health Organisation disability assessment scale. (PDF 24 kb)

Additional file 5: Physiotherapist ward data collection and protocol form. Case report form for ward physiotherapists. (PDF 403 kb)

Additional file 6: ICEAGE Data Dictionary. Data fields for ICEAGE. (DOCX 18 kb)

## Abbreviations

CRF: Case report form
CXR: Chest X-ray
DB&C: Deep breathing and coughing
EQ-5D: EuroQual five domains
ICEAGE: Incidence of Complications following Emergency Abdominal surgery—Get Exercising
ICU: Intensive care unit
LOS: Length of stay
mILOA: Modified Iowa level of assistance
PPC: Postoperative pulmonary complication
PPOI: Prolonged postoperative ileus
QOL: Quality of life
SAE: Serious adverse event
SAQ: Self-administered physical activity questionnaire
SOFA: Sequential organ failure assessment
VAS: Visual analogue scale
WHODAS: World Health Organisation disability assessment score
