# Supplementary material for: ICEAGE (Incidence of Complications following Emergency Abdominal surgery: Get Exercising): study protocol of a pragmatic, multicentre, randomised controlled trial testing physiotherapy for the prevention of complications and improved physical recovery after emergency abdominal surgery
Source: World J Emerg Surg. 2018 Jul 3;13:29. doi: 10.1186/s13017-018-0189-y (PMC6029354; doi:10.1186/s13017-018-0189-y)
Supplement: Supplementary file 1 — Postoperative Physiotherapy Discharge Scoring Tool. Table describing the discharge from physiotherapy scoring. (DOCX 15 kb) [file 13017_2018_189_MOESM1_ESM.docx]

**THE MODIFIED IOWA LEVEL OF ASSISTANCE (ILOA) SCALE**

**TASKS**

- Supine to sitting on the edge of the bed
- Sitting on the edge of the bed to standing
- Walking
- Negotiation of 1 step
- Walking distance
- Assistive device use

Each task is graded according to the level of assistance required (for supine to sit, sit to stand, walking and negotiation of 1 step); walking distance completed and gait aid used for mobility tasks. It ranges from 0-36 points

**ORDINAL SCALE AND DEFINITIONS FOR LEVEL OF ASSISTANCE**

**0** – Independent No assistance or supervision is necessary to safely perform the activity with or without assistive devices, aids or modifications

**1** – Standby Nearby supervision is required for safe performance of activity; no contact is necessary

**2** – Minimal One point of contact is necessary for the safe performance of the activity including helping with the application of the assistive device (part of ambulation), getting leg(s) on or off the leg rest and stabilising an assistive device

**3** – Moderate Two points of contact are necessary (by one or two persons) for the safe performance of the activity

**4** – Maximal Significant support is necessary at a total of three or more points of contact (by one or more people) for the safe performance of the activity

**5** – Failed Attempted activity but failed with maximal assistance

**6** – Not tested Due to medical reasons or for reasons of safety, test was not attempted

**Contact** Any physical contact between therapist and the patient or the assistive device (frame, crutches etc)

**ORDINAL SCALE FOR WALKING DISTANCE**

1. >40 metres
2. 26-40 metres
3. 10-25 metres
4. 5-9 metres
5. 3-4 metres
6. 2 metres
7. <2 metres

**ORDINAL SCALE FOR USE OF ASSISTIVE DEVICE**

This score is given based on assistive device required for sit to stand, walking and step.

1. No assistive device
2. One stick or crutch
3. Two sticks
4. Two elbow crutches
5. Two axillary crutches
6. Frame (standard or wheely)

Gutter, platform frame, standing lifter, hoist, or it is deemed not safe to use a frame
