# Supplementary material for: ICEAGE (Incidence of Complications following Emergency Abdominal surgery: Get Exercising): study protocol of a pragmatic, multicentre, randomised controlled trial testing physiotherapy for the prevention of complications and improved physical recovery after emergency abdominal surgery
Source: World J Emerg Surg. 2018 Jul 3;13:29. doi: 10.1186/s13017-018-0189-y (PMC6029354; doi:10.1186/s13017-018-0189-y)
Supplement: Supplementary file 2 — EQ-5D-5L. EuroQual 5 Domains scoring. (DOCX 90 kb) [file 13017_2018_189_MOESM2_ESM.docx]

|  |
| --- |
| Health Questionnaire |
|  |
|  |
| English version for Australia |

| Under each heading, please tick the ONE box that best describes your health TODAY. | |
| --- | --- |
| MOBILITY |  |
| I have no problems with walking around | ❑ |
| I have slight problems with walking around | ❑ |
| I have moderate problems with walking around | ❑ |
| I have severe problems with walking around | ❑ |
| I am unable to walk around | ❑ |
| PERSONAL CARE |  |
| I have no problems with washing or dressing myself | ❑ |
| I have slight problems with washing or dressing myself | ❑ |
| I have moderate problems with washing or dressing myself | ❑ |
| I have severe problems with washing or dressing myself | ❑ |
| I am unable to wash or dress myself | ❑ |
| USUAL ACTIVITIES *(e.g. work, study, housework, family or leisure activities)* |  |
| I have no problems doing my usual activities | ❑ |
| I have slight problems doing my usual activities | ❑ |
| I have moderate problems doing my usual activities | ❑ |
| I have severe problems doing my usual activities | ❑ |
| I am unable to do my usual activities | ❑ |
| PAIN / DISCOMFORT |  |
| I have no pain or discomfort | ❑ |
| I have slight pain or discomfort | ❑ |
| I have moderate pain or discomfort | ❑ |
| I have severe pain or discomfort | ❑ |
| I have extreme pain or discomfort | ❑ |
| ANXIETY / DEPRESSION |  |
| I am not anxious or depressed | ❑ |
| I am slightly anxious or depressed | ❑ |
| I am moderately anxious or depressed | ❑ |
| I am severely anxious or depressed | ❑ |
| I am extremely anxious or depressed | ❑ |

The best health you can imagine

| We would like to know how good or bad your health is TODAY. |
| --- |
| This scale is numbered from 0 to 100. |
| 100 means the best health you can imagine. 0 means the worst health you can imagine. |
| Mark an X on the scale to indicate how your health is TODAY. |
| Now, please write the number you marked on the scale in the box below. |

YOUR HEALTH TODAY =

10

0

20

30

40

50

60

80

70

90

100

5

15

25

35

45

55

75

65

85

95

The worst health you can imagine
