# Supplementary material for: ICEAGE (Incidence of Complications following Emergency Abdominal surgery: Get Exercising): study protocol of a pragmatic, multicentre, randomised controlled trial testing physiotherapy for the prevention of complications and improved physical recovery after emergency abdominal surgery
Source: World J Emerg Surg. 2018 Jul 3;13:29. doi: 10.1186/s13017-018-0189-y (PMC6029354; doi:10.1186/s13017-018-0189-y)
Supplement: Supplementary file 3 — WHODAS. World Health Organisation disability assessment scale. (DOCX 20 kb) [file 13017_2018_189_MOESM3_ESM.docx]

Time point: 1 year from surgery Date: ___________

**12-item version, self-administered**

This questionnaire asks about difficulties due to health conditions. Health conditions include diseases or illnesses, other health problems that may be short or long lasting, injuries, mental or emotional problems, and problems with alcohol or drugs.

Think back over the past 30 days and answer these questions, thinking about how much difficulty you had doing the following activities. For each question, please circle only one response.

| In the past 30 days, how much difficulty did you have in: | | | | | | |
| --- | --- | --- | --- | --- | --- | --- |
|  |  | None | Mild | Moderate | Severe | Extreme /cannot do |
| S1 | Standing for long periods such as 30 minutes? | 1 | 2 | 3 | 4 | 5 |
| S2 | Taking care of your household responsibilities? | 1 | 2 | 3 | 4 | 5 |
| S3 | Learning a new task, for example, learning how to get to a new place? | 1 | 2 | 3 | 4 | 5 |
| S4 | How much of a problem did you have joining in community activities (eg, festivities, religious or other activities) in the same way as anyone else can? | 1 | 2 | 3 | 4 | 5 |
| S5 | How much have you been emotionally affected by your health problems? | 1 | 2 | 3 | 4 | 5 |
| S6 | Concentrating on doing something for ten minutes? | 1 | 2 | 3 | 4 | 5 |
| S7 | Walking a long distance such as a kilometre [or equivalent]? | 1 | 2 | 3 | 4 | 5 |
| S8 | Washing your whole body? | 1 | 2 | 3 | 4 | 5 |
| S9 | Getting dressed? | 1 | 2 | 3 | 4 | 5 |
| S10 | Dealing with people you do not know? | 1 | 2 | 3 | 4 | 5 |
| S11 | Maintaining a friendship? | 1 | 2 | 3 | 4 | 5 |
| S12 | Your day to day work? | 1 | 2 | 3 | 4 | 5 |

Have you had any new illness, injury, or health problem in the last 6 months? YES / NO

If yes, when did it happen? Date: ________________

Anything else you would like to let us know?

This completes the questionnaire. Thank you.
