# Supplementary material for: ICEAGE (Incidence of Complications following Emergency Abdominal surgery: Get Exercising): study protocol of a pragmatic, multicentre, randomised controlled trial testing physiotherapy for the prevention of complications and improved physical recovery after emergency abdominal surgery
Source: World J Emerg Surg. 2018 Jul 3;13:29. doi: 10.1186/s13017-018-0189-y (PMC6029354; doi:10.1186/s13017-018-0189-y)
Supplement: Supplementary file 4 — WHODAS. World Health Organisation disability assessment scale. (PDF 24 kb) [file 13017_2018_189_MOESM4_ESM.pdf]

HOW TO CALCULATE VO_2 USING SELF ADMINISTERED ACTIVITY QUESTIONNAIRE (SAQ) SCORE:

Step 1: Instruct patient to complete SAQ

Step 2: Determine the individual, highest MET value for that patient to calculate their SAQ according to the following table: *e.g. if the patients highest scoring question was question* **e.** *then their SAQ=7.00*

Can you complete the following without symptoms?	***MET value***
**m.** Dress without stopping because of symptoms?	2.00
**j.** Do moderate work around the house like vacuum, sweep floors, or carry groceries?	2.50
**a.** Walk down a flight of stairs unassisted and without stopping?	3.00
**k.** Do heavy work around the house like strip and make the bed, hang out washing, or wash the car?	3.25
**c.** Do moderate gardening like weed or rake the leaves?	4.25
**l.** Push an electric or petrol mower on level ground?	4.50
**h.** Participate in moderate activities like walk at a normal pace (4km/hr) or play golf and carry the clubs?	4.75
**d.** Walk briskly around an oval?	5.00
**g.** Do outdoor work like split wood or dig in the garden?	5.50
**b.** Carry an 8kg weight (e.g. a load of wet washing) up 8 steps?	6.00
**e.** Carry at least 10kg (e.g. a suitcase) up 8 steps?	7.00
**f.** Carry objects that weigh at least 35kg (e.g. an 11 year old child)?	7.50
**i.** Participate in vigorous activities like swimming (crawl), jogging (8km/hr), cycling (17km/hr) or singles tennis?	9.00

MET = metabolic equivalent

Step 3: Record the patient’s age in years, height in centimetres and weight in kilograms.

Step 4: To calculate VO_2; substitute these values into the following equation:

$$VO_2 = 2.36(SAQ) + 0.35(HEIGHT\ cm) - 0.19(AGE\ yrs) - 0.16(WEIGHT\ kg) - 33.89$$

SELF-ADMINISTERED PHYSICAL ACTIVITY QUESTIONNAIRE (SAQ)

Can you complete the following activities at a normal rate?
***Circle* YES *or* NO**

		*
**a. Walk down a flight of stairs unassisted and without stopping?**	**YES**	**NO**
**b. Carry an 8kg weight (e.g. a load of wet washing) up 8 steps?**	**YES**	**NO**
**c. Do moderate gardening like weed or rake the leaves?**	**YES**	**NO**
**d. Walk briskly around an oval?**	**YES**	**NO**
**e. Carry at least 10kg (e.g. a suitcase) up 8 steps?**	**YES**	**NO**
**f. Carry objects that weigh at least 35kg (e.g. an 11 year old child)?**	**YES**	**NO**
**g. Do outdoor work like split wood or dig in the garden?**	**YES**	**NO**
**h. Participate in moderate activities like walk at a normal pace (4km/hr) or play golf and carry the clubs?**	**YES**	**NO**
**i. Participate in vigorous activities like swimming (crawl), jogging (8km/hr), cycling (17km/hr) or singles tennis?**	**YES**	**NO**
**j. Do moderate work around the house like vacuum, sweep floors, or carry groceries?**	**YES**	**NO**
**k. Do heavy work around the house like strip and make the bed, hang out washing, or wash the car?**	**YES**	**NO**
**l. Push an electric or petrol mower on level ground?**	**YES**	**NO**
**m. Dress without stopping because of symptoms?**	**YES**	**NO**

*** If you answered "NO" to any of the above questions, what stops you doing these activities? (e.g. shortness of breath, angina, recent surgery etc.)**

____

____

____
