# Supplementary material for: ICEAGE (Incidence of Complications following Emergency Abdominal surgery: Get Exercising): study protocol of a pragmatic, multicentre, randomised controlled trial testing physiotherapy for the prevention of complications and improved physical recovery after emergency abdominal surgery
Source: World J Emerg Surg. 2018 Jul 3;13:29. doi: 10.1186/s13017-018-0189-y (PMC6029354; doi:10.1186/s13017-018-0189-y)
Supplement: Supplementary file 5 — Physiotherapist ward data collection and protocol form. Case report form for ward physiotherapists. (PDF 403 kb) [file 13017_2018_189_MOESM5_ESM.pdf]

ICEAGE TRIAL (V8 June 2017) - ward physiotherapist sheet
POST OPERATIVE DATA COLLECTION

Patient sticker or name

Intervention Group	Control Group
**Treatment Protocol:**	**Treatment Protocol:**
Chest physio - min **twice daily coached for the first 2 days, then ongoing as necessary**	Chest physio - **once only on the first day**
direct coaching of 10 reps DB with 2-5 sec insp holds per rep, repeat 2 sets	direct coaching of 10 reps DB with 2-5 sec insp holds per rep, repeat 2 sets
instruct patient to perform 10x2 sets every hour self-directed	instruct patient to perform 10x2 sets every hour self-directed
provide cough pillow, give info booklet	provide cough pillow, give info booklet
daily reminder first 5 days to continue DB&C exercises. Repeat coached session if necessary	No further chest physio or reminders
Rehab physio - **at least 30mins every day with a PT for at least first 5 days**	Rehab physio - **ambulation protocol once daily until d/c score=14 or 15**
Rehab Rx continues after day 5 until patient reaches physio d/c score >13	Tell pt to ambulate as often as able.
Rehab Rx (as per protocol) can be delegated to AHA after day 5 if appropriate	Can handover patient to AHA once patient amb>Stage 3 and safe
Provide rehab as per scale in sequence i.e attempt ambulation >15min	Ward physio to assess patient daily for d/c from services using screening tool
If unable to ambulate, attempt sit to stand, and so on.	AHA to screen patient for safety prior to ambulation using screening tool
Total exercise time is combined work time of all exercises performed	Provide ambulation assistance until patient reaches physio d/c score of 14 or 15
Not including time resting or not moving	
**Exercise Scale**- MUST provide AT LEAST 30minutes total**	**Ambulation protocol#: NO MORE THAN 15 mins of ambulation**
**1. Ambulation as per control group ambulation protocol aim>15mins**	**1. (safety) Sitting min 2 min**
**2. Sit to stand – raised bed progressed to ward chair - Low resistance, 50% 8-10RM. Can include step ups**	**2. (safety) MOS 0-1 min**
	**3. (Amb) MOS/walk 1-3 mins**
**3. UL or LL in sitting – against gravity progressed to theraband resisted - Low resistance, 50% 8-10RM. Can include seated pedals**	**4. (Amb) MOS/walk 3-6 mins**
	**5. (Amb) Walk 6-10 mins**
**4. Sit over edge of bed - pt to support selves as much as able**	**6. (Amb) Walk 10-15 mins**
	**7. (Amb) Walk >15 mins**
**5. Bed exercises (eg, bridging, slide boards, active assisted ROM)**	**Goals** - increase RR, **RPE 3-4/10**, aim for 10mins (Stage 6) of total walk time
**6. Deep breathing and coughing exercises**	Intervals of equal work:rest time allowable to achieve 10mins total work time
**7. Passive mobilisation (eg passive cycling, FES, passive ROM)**	Record reason if unable to achieve Stage 6

FAQS

What do I do if a patient gets diagnosed with a PPC? — Treat the patient with chest physiotherapy as you see fit (i.e DB&C, PEP). **Continue treating as per rehab protocol.**

What do I do if a patient is determined to require formal rehab services or a surg team requests further physio input due to mobility dysfunction or slow progess? — Contact site investigator. Normally this would mean that the patient can stop being treated according to the protocol and started with a patient specific rehab program

Everyday, BEFORE seeing patient determine d/c status. If score >13: CONTROL - no physio required. INTERVENTION - continue as per protocol until POD6, then d/c from PT once score>13

**Discharge from Physiotherapy scoring (d/c when score is 14 or 15)**							
**Date**							
low score = worse	**DAY 1**	**DAY 2**	**DAY 3**	**DAY 4**	**DAY 5**	**DAY 6**	**DAY 7**
Mobility (0-3)							
Breath Sounds (1-3)							
Secretion clearance (1 **OR** 3)							
SpO_2 (2 ,3)							
Resp rate (2, 3)							
**D/C from Physio (6-15)**							
**Ambulation: INTERVENTION (every day for 5 days and cont till d/c from PT), CONTROL GROUP (daily until d/c from PT score >13)**							
Who provided it? Name and profession							
Time of day (24hr clock time)							
Pain score pre amb (0-10)							
Mobility stage attained# (1-7)							
Reason for not achieving Stage 6 (1-8)							
Max Borg (0-10)							
**Chest Physiotherapy: INTERVENTION (at least twice daily for first 2 days, continue as neccessary), CONTROL (once only on first day)**							
Who provided it? Name and profession							
Time of day (24hr clock time)							
Number of sessions							
**Rehabilitation Exercises: INTERVENTION ONLY (every day with PT for 5 days and then on till d/c from PT)**							
Highest level achieved (exercise scale** 1-7)							
Total treatment session duration (mins)							
If <30mins, reason for not achieving 30min							
Details							

**Document any adverse events (+ call site PI) or breaks to protocol (i.e you treat a control pt with more than 15 mins of exercise!!)**							
Adverse event (see over for list)							
Breaks to protocol (see over for list)							

Physio Discharge Criteria	
Mobility	3 = Reached pre-op ambulation status<br>2 = Requires supervision, status has plateaued<br>1 = Requires assistance, status is improving<br>0 = Unable to ambulate
Breath Sounds	3 = Reached pre-op levels and within expectations for patient<br>2 = Slightly decreased BS or presence of a few added sounds<br>1 = Markedly abnormal BS and/or significant added sounds
Secretion clearance	3 = able to clear secretions indep OR at pre-op status<br>1 = Requires assistance to clear secretions
SpO2 on Room Air<br>Remove O_2 for up to 2mins	3 = Sats >92% or >88% (existing resp condition)<br>2 = Sats <92% or <88% (existing resp condition)
Resp rate	3 = Within normal expectations<br>2 = Outside acceptable range for the individual

Break to protocol	
1	Patient informs assessor of group
2	Treat a patient incorrectly or not to
3	Other (specify)

Reason for not ambulating as per protocol	
1	Hypotension (dizzy BP<100/60, in sitting after 2 min ankle pumping and rest)
2	Pain (> 7/10, analgesia active, distressed)
3	Nausea, vomiting
4	Patient unavailable
5	Physio/assist unavailable
6	Patient non consent
7	Other (specify)
8	Fatigue

Adverse event - report in notes & advise PI ASAP	
1	BP change 20% from resting
2	HR change 20% from resting
3	New arrhythmia
4	Drop in SpO2 >10%
5	PAP > 60 mmHg
6	Pneumothorax following intervention
7	Line detachment
8	Patient requires increased sedation
9	Patient requires increased inotropic support
10	Fall
11	Severe nausea
12	Other (specify)

Documentation:

To maintain blinding of the assessor, any **treatment documentation must** be kept separate from the main medical record **until Day 7 or until PPC dx. (Both intervention and control treatment notes are to be kept seperate)**. At this point the physio Rx notes can be reintegrated within the medical record and the assessor can become unblinded.

All patients in the trial get chest physio on the first day. This session can be documented in the medical record. However, the 2nd chest physio treatment session for the intervention group must be recorded in the separated notes.

Record in the medical record daily (during the period of physio Rx) any assessment details including respiratory status and the level of assistance required for the following tasks, according to the standardised rating below.

Document the three below tasks as either independent, standby assist, min assist, mod assist, max assist, failed, or not assessed. Use the definitions provided.

**SOEOB**	Independent	No assistance or supervision is necessary to safely perform the activity with or without assistive devices or aids
**Sit to stand**	Standby assist	Nearby supervision is required for safe performance of activity; no contact* is necessary
**Mobility**	Minimal assist	One point of contact* is necessary for the safe performance of the activity
	Moderate assist	Two points of contact* are necessary (by 1 or 2 persons) for the safe performance of the activity
	Maximal assist	Significant support is necessary at a total of 3 or more points of contact* (by 1 or more) for safe performance
	Failed	Attempted activity but failed with max assistance
	Not assessed	Due to medical reasons or for reasons of safety, test was not attempted
		*Contact = any physical contact between therapist and the patient or assistive device (i.e walking aid)
**Walking distance**	Record the estimated maximum walking distance (but **NOT** the time) achieved by the patient in the physio or AHA session	
**Mobility aid**	State the mobility aid being used each day eg hoist, standing hoist, FASF, 4WF	

Example of an ICEAGE patient documentation

PHYSIOTHERAPY - HDU

12/01/2017 | 11:00 | HDU

58 y.o male DAY 1 emergency laparotomy for a perforated duodenal ulcer.

PHx: COPD, diabetes, BMI>30, OA left hip

SHx: Unemployed, lives alone

Mobility Hx: Indep amb no mobility aid. SOBOE after approx 200m flat. SOBOE hills and stairs. Limited by SOB, prior to pain in L hip.

Resp Hx: Current smoker (started 15y.o, 30 per day = 50 pack years)
Daily productive cough - 1-2 tsp of white phlegm
No recent chest infection

Currently, RIB and drowsy. No nausea

Resp: SpO2 98% on Airvo 40% at 40LPM via HFNP. SpO2 ↓ 91% on RA
ausc - ↓ AE bibasal + fine end inspiratory crackles RLL
cough - weak, moist, productive of white sputum, strength limited by pain

D&D - HFNP, IV x 2, IDC, abdo drain x 2, NGT

CVS: BP normotensive, no vasopressors. HR 80

Pain: 2/10 at rest. 6/10 with cough and movt in bed.
Analgesia - PCA

Mobility: SOEOB - mod A x 2
sit to stand - mod assist x 1
mob - mod A x 2 approximately 50m with FASF + IV Pole. Limited by pain. Stairs not assessed.

Rx: Coached DB&C exercises 10x2 with inspiratory holds. Educated to continue to perform every hour.
Mobilised as per ICEAGE trial protocol - max RPE 4/10, no adverse events

Plan: Progress mobility. If pain continues to significantly limit mobility or respiratory exercises raise with Pain team.

Key components for ICEAGE Ax doc
- SpO_2 on oxygen **and** on RA
- Auscultation
- Cough Ax including colour phelgm
- Assistance required for SOEOB, Sit to stand, mobility
- Walking distance
- Aid used
- Stair assessed?
- max RPE during mobility
- adverse events?

If unable to mobilise as per protocol, state reason why.

For **all** patients, regardless of group, record in
Separate notes: duration of activity session (minutes)
Med record: distance (metres up to 100m) or "pt amb >100m"

Note:

INTERVENTION GROUP - record all ongoing chest treatments and rehab exercises in separated notes

CONTROL GROUP - Physio to document in the medical record resp and mobility status (can take from nurse report and obs) for the first 5 days, even if d/c from physio treatment.
If patient being seen by AHA within the first 5 PODs, these Rx notes also need to be entered in the separated notes.
