# Supplementary material for: ICEAGE (Incidence of Complications following Emergency Abdominal surgery: Get Exercising): study protocol of a pragmatic, multicentre, randomised controlled trial testing physiotherapy for the prevention of complications and improved physical recovery after emergency abdominal surgery
Source: World J Emerg Surg. 2018 Jul 3;13:29. doi: 10.1186/s13017-018-0189-y (PMC6029354; doi:10.1186/s13017-018-0189-y)
Supplement: Supplementary file 6 — ICEAGE Data Dictionary. Data fields for ICEAGE. (DOCX 18 kb) [file 13017_2018_189_MOESM6_ESM.docx]

**DATA DICTIONARY**

Baseline Characteristics

| Parameter | Method |
| --- | --- |
| Gender | Male or Female |
| Age | Age in years |
| Body Mass Index | weight (kg) / height (m) squared |
| Surgical category | 1. Hepatobiliary and Upper Gastrointestinal  2. Colorectal and lower Gastrointestinal  3. Renal & Urology  4. Other |

**Group comparison/PPC risk analysis/general data points**

| Co-morbidities | Functional Co morbidity Index (0-18 scale)  Including respiratory disease, cancer, diabetes, cardiovascular disease, peripheral vascular disease, musculoskeletal, neuromuscular disease |
| --- | --- |
| Smoking history | 1. Non smoker  2. Current smoker  3. Ex smoker (ceased >8weeks pre-operatively) |
| Pack years | 1 pack year = 20 cigarettes per day for 1 year |
| Pre-op health status | ASA from anaesthetic record |
| Anaesthesia duration | Time in minutes |
| Incision type | 1. Midline laparotomy  2. Bilateral subcostal (Chevron)  3. Subcostal (Kocher)  4. Transverse  5. Abdominal incision  6. Other  7. Mini-laparotomy |
| Type of post-operative analgesia | 1. Epidural  2. Constant opioid infusion  3. Patient controlled analgesia  4. Patient controlled epidural analgesia  5. Oral  6. Other |
| Length of analgesia | Number of post-op days |
| ICU admission | Yes/no |
| Length of ICU stay | Length in days |
| Mechanical ventilation | Hours |
| POD of diagnosed PPC | POD |
| Incidence of death | Yes/no |
| Reason for withdrawal from trial | 1. Withdrew self from trial 2. Does not consent after having been randomised |

Post-operative measurements

**PPC detection**

| CXR report of collapse/consolidation | Yes/no/not available |
| --- | --- |
| Minimum daily SpO_2_ on room air | One measure daily from observation chart |
| Maximum daily tympanic temperature | One measure daily from observation chart |
| WCC > 11 OR AB for chest infection | Yes/no/not available |
| Sputum presence | Daily measure, yes/no  Defined as production of green or yellow sputum different to pre-operative assessment or reported by the patient. |
| Bacteria growth shown in sputum sample | Yes/no/not available |
| Auscultation abnormal findings | Daily measure yes/no  Defined as new abnormal breath sounds different to pre-operative auscultation as documented by Dr or assessing physiotherapist |
| Dr diagnosis of PPC | Yes/no |

**PPOI detection**

| Nausea or vomiting | Yes/no |
| --- | --- |
| Inability to tolerate oral diet last 24hrs | Yes/no |
| Absence of flatus last 24hrs | Yes/no |
| Abdominal distention | Yes/no |
| Radiologic confirmation | Yes/no/not available |
| Doctors diagnosis of ileus | Yes/no |

**Outcome measures**

| Hospital length of stay | Days |
| --- | --- |
| Time to readiness to discharge | Day post-op, using Fiore tool or modified version for surgical categories other than colorectal |
| POP DST | Score out of 15 assessed POD 1,2,3,4,5. If discharge from physiotherapy not achieved by POD 5, continue daily assessment until discharged from Physiotherapy. |
| Time to discharge from physio | Day post-op, using POP DST |
| Incidence of pulmonary complications | Yes/no (using defined diagnostic criteria) |
| Incidence of prolonged post-op ileus | Yes/no (using defined diagnostic criteria) |
| Recovery of strength | Grip strength in kgs taken POD 1, 3, 5, 7, Day of d/c. This measurement should ideally be performed within the same time period within +/- 3 hours of the baseline measurement. |
| Functional ability | Score on the Modified Iowa Level of Assistance (MILOA) Scale (See Appendix D) taken between 1 and 4pm on POD 1, 3, 5, 7, and on d/c |
| Medium term recovery | Quality of Recovery (QoR-15) (See Appendix E) assessed POD 1 & 3 and at 3 months from surgery WHODAS-12 disability questionnaire (See Appendix F) assessed day of discharge and at 3 months. |
| Complications – self report at 3 months post-surgery | Hospitalisation following discharge, hospital visit or GP visit for complications including  1. PE/DVT  2. Respiratory  3. Cardiac  4. Surgical/wound complication  5. Ileus/constipation/nausea  6. Fatigue/tiredness/weakness  7. Death |
| Discharge destination | 1. Home 2. Rehab facility 3. Nursing home 4. Other hospital |

**Cost analysis/health care utilisation**

| Days of supplemental oxygen usage | Days |
| --- | --- |
| Hours of mech vent/NIV usage | Hours |
| Occasions of service by a physio | Number of treatment sessions |
| Occasions of service by a PTA | Number treatment sessions |
| Extra-ordinary attendance by MO | Number of occasions |
| Incidence of non-respiratory complications | For example, sepsis, DVT, PE, surgical failure, cardiovascular event. Event reason documented and POD occurred |
| Intra and post-operative fluid delivery | Type and amount |
| Intra and post-operative blood products  Clinical coding | Type and amount |

**Daily ambulation sessions**

| Time of day | Time |
| --- | --- |
| Mobility level achieved each POD | 1. SOEOB 0-2 minutes  2. MOS 0-1 minute  3. MOS/walk 1-3 minutes  4. MOS/Walk 3-6 minutes  5. Walk 6-10 minutes  6. Walk 10-15 minutes  7. Walk > 15 mins |
| Max Borg scale per physio session | 1-10 scale |
| Barriers to mobilisation |  |
| Symptomatic hypotension limiting ambulation | Yes/no daily  Defined: sitting BP<100/60, dizzy, and not relieved through 2 mins of ankle pumping whilst sitting |
| Incidence of vomiting limiting mobilisation | Yes/no daily |
| Pain score prior to physio ambulation | 10 point verbal scale daily |
